# What are the metabolic precursors which increase the risk of pre-eclampsia and how could these be investigated further

**DOI:** 10.1016/j.placenta.2017.08.011

**Published:** 2017-12

**Authors:** Jenny E. Myers

**Affiliations:** a Maternal & Fetal Health Research Centre, Division of Developmental Biology and Medicine, School of Medical Sciences, Faculty of Biology, Medicine and Health, University of Manchester, Manchester Academic Health Science Centre, UK; b St Mary's Hospital, Central Manchester Foundation Trust, Manchester Academic Health Science Centre, Manchester, M13 9PL, UK

## Abstract

Several maternal and pregnancy characteristics have been associated with an increased risk of preeclampsia in epidemiological studies. This review discusses metabolic risk factors in particular and their interaction with other maternal and/or pregnancy characteristics. Examples of research studies that have used data from women with specific characteristics or explored the interaction between risk factors are discussed. Suggestions for future research using large data sets and incorporating knowledge of cardiovascular disease and other metabolic diseases are also highlighted.

## Highlights

• Obesity and nulliparity account for the largest population risk of preeclampsia.
• Low PlGF in early pregnancy is more strongly associated with preeclampsia in obese women.
• There are interactions between obesity and other characteristics which amplify the risk of preeclampsia e.g. ART, ethnicity.

## 1 Introduction

Pre-eclampsia continues to be a leading cause of maternal and neonatal mortality and morbidity affecting 3-8% of pregnancies worldwide. Exact information on the worldwide incidence of the condition and temporal changes in incidence are not available from many countries. However, data from the USA has suggested that rates have increased in recent years; 2.4% between 1987-8 to 2.9% in 2003-4 [1]. An increase was also reported in a Norwegian data set which documented rates of 3.7% between 1988 and 1992 and 4.4% between 1998 and 2002 [2]. There are many risk factors for the development of preeclampsia described in the literature, these include a prior history of gestational hypertensive disease, nulliparity, family history, obesity, pre-existing medical disease, primipaternity, assisted reproduction and short duration of sperm exposure and extremes of maternal age [3]. The metabolic health of women of reproductive age has changed over the last few decades, such that obesity is now one of the most important risk factors for the development of preeclampsia. Moreover, assisted reproductive techniques have advanced dramatically over the same time period. This review will focus on the metabolic risk factors associated with preeclampsia and discuss what is already known and suggest potential avenues for future research.

There are many cohort studies which have quantified the risk associated with the development of preeclampsia and these have recently been synthesised by Bartsch et al. [4] in a recent meta analysis in which 25,356,688 women from 40 studies in Europe and 30 studies in North America. Previous gestational hypertensive disease, chronic hypertension and antiphospholipid syndrome were demonstrated to be associated with the highest absolute risk. However, in terms of population attributable risk, obesity and nulliparity accounted for the largest population risk. Similar data have also been collated from low and middle income settings with data from 276,388 mothers and their infants analysed by investigators at the World Health Organisation [5]. The prevalence of preeclampsia/eclampsia in this study population was 4% and the odds ratio for development of the condition associated with BMI ≥ 35, nulliparity and chronic hypertension were 3.90 [3.52–4.33], 2.04 [1.92–2.16] and 7.75 [6.77–8.87], respectively. This study confirms that across disparate geographical locations these risk factors appear to have the greatest impact on the risk of preeclampsia.

**Fig. 1:**
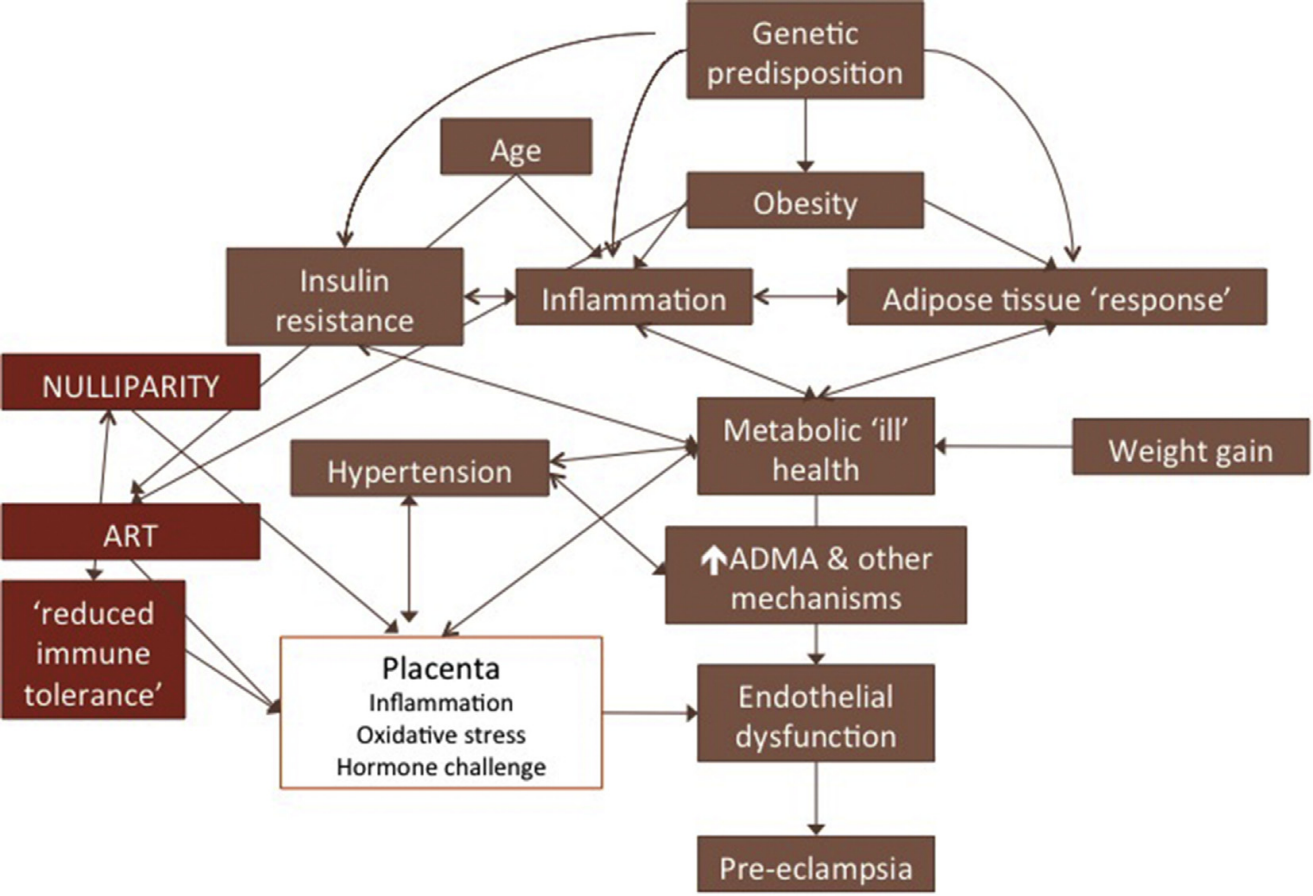
Metabolic precursors for preeclampsia and their interactions. This figure illustrates the interaction between the metabolic precursors to preeclampsia and their interaction with other components of the causative pathway. The figure is not intended to be comprehensive and includes only some of the major interactions.

The potential interplay between several risk factors and preeclampsia is illustrated in Fig. 1. Whilst the identification of risk factors for pre-eclampsia has led to numerous avenues of research and hypothesis generation, it is frustrating that these epidemiological observations, which have been very consistently reported, have not led to major breakthroughs in our understanding of the condition. Despite the consistent associations between the risk factors and the development of preeclampsia, specific causative associations remain poorly understood. The absence of a definitive causative link is attributable, in part, to the fact that the number of women with any given risk factor not affected by the condition will always outweigh the number who will be affected. This was exemplified in the SCOPE cohort in which 5690 healthy nulliparous women were recruited [6]. Women with a BMI ≥30 kg/m^2^ were twice as likely to develop preeclampsia, however the number of women with a BMI≥ 30 kg/m^2^ who did not develop preeclampsia outweighed those with the disease by 10:1. In addition, estimating an individual woman's risk from the epidemiological data is currently not possible from the cohort data available, as despite their frequent coexistence in clinical practice, the potential multiplicative effect of several risk factors has rarely been considered in cohort studies [4]. What can be learnt from these epidemiological risk factors, which might progress our understanding of the origins of this heterogeneous syndrome?

## 2 Obesity

Of the factors associated with preeclampsia obesity has been the most thoroughly studied with at least some attention to potential mechanisms. In this review it will serve as a model for the approach to understand the mechanisms associated with other risk factors.

### 2.1 Epidemiological considerations

The burden of obesity is increasing globally with many countries now having more than a third of adults and a fifth of adolescents classified as obese. Obesity is the leading attributable risk factor for the development of preeclampsia and appears to incur a dose-dependent relationship with the risk of developing preeclampsia with a continued increase in risk with higher categories of BMI [7]. In a large population study in Missouri, there was an incremental increase in the risk of preeclampsia with increasing BMI [8]. Whilst there was an increased risk of both early and late preeclampsia, the association between obesity and late preeclampsia was stronger. Different risks in women with equivalent BMIs have also been reported in women of different ethnicities [7].

### 2.2 Interaction between obesity and other biomarkers

Obesity is a risk factor for both cardiovascular disease and preeclampsia [9] and it is likely that the common risk features include components of the metabolic syndrome: hypertension, insulin resistance and dyslipidaemia [10]. In addition, obesity is likely to contribute to the pathophysiology of preeclampsia through altered inflammatory profiles [11]. It is has been estimated that around 30% of the association between preeclampsia and obesity is mediated through abnormal inflammatory profiles signified by elevated *C*-reactive protein (CRP) levels, an inflammatory mediator produced by the liver as well as adipocytes and implicated in cardiovascular morbidity [11], [12]. Research studies such as the study by Bodnar et al. [11], which aimed to dissect out the key pathways relevant for the development of preeclampsia in obese women may progress the research field more quickly than studies which include women with a multitude of different risk factors. Another example of such a study was performed within the SCOPE cohort and interrogated biomarker profiles in obese women who subsequently developed preeclampsia compared to normal weight individuals [13]. A number of predictors were different between women in the different BMI groups. For example, blood pressure in early pregnancy was more strongly associated with preeclampsia in women with normal BMI than in those with obesity, in whom it was consistently raised independent of pregnancy outcome. Another key finding was that Placental growth factor (PlGF), a member of the vascular endothelial growth factor (VEGF) family, was more strongly predictive of preeclampsia in obese women who developed preeclampsia than their normal weight counterparts. In the full SCOPE cohort, low PlGF was a predictor for preeclampsia but only in women with preterm disease [6]. The stronger association between low PlGF in early pregnancy and preeclampsia in obese women is particularly intriguing as the majority of cases of preeclampsia among women with obesity were near term [14]. One possible explanation for this observation is that low PlGF in women with obesity may be a feature of adiposity, rather than placental function, and attributable to the effects of adipokines on extraplacental sites of synthesis [15] such as the vascular endothelium. Other studies have also identified abnormal angiogenic profiles in obese women. Lower levels of sFlt-1 and PlGF [16], lower levels of PlGF [17] and higher a sFlt-1/PGF ratio [18] have all been reported in the context of obesity and preeclampsia, indicating an anti-angiogenic milieu even in early pregnancy. Abnormal levels of the adipokines, leptin and adiponectin have also been implicated in cardiovascular disease, obesity [19] and preeclampsia [20] [21]. Furthermore, abnormal angiotensinogen production from adipocytes may contribute to abnormal vascular function in obese women through activation of the renin-angiotensin system [22] and increased production of free fatty acids may contribute to increased oxidative stress. Abnormal adipocyte function therefore offers a plausible component of the link between obesity and preeclampsia risk and further research in this area is essential.

Increased oxidative stress has long been linked to obesity and preeclampsia and lower levels of circulating antioxidants have been demonstrated in obese individuals who are not pregnant [23]. Whilst trials of antioxidants have not yielded positive results for the prevention of preeclampsia, evidence of increased oxidative stress both within the maternal vasculature and within the placenta are still thought to contribute the pathophysiology of preeclampsia [24], [25]. Another key pathway linking abnormal vascular function and obesity [26], and therefore potentially preeclampsia, is nitric oxide (NO) bioavailability (see Box 1). Increased concentrations of asymmetric dimethylarginine (ADMA), an endogenous inhibitor of nitric oxide (NO) synthase, is a risk factor for cardiovascular disease and is associated with inflammation, insulin resistance and dyslipidaemia [26]. Furthermore, circulating ADMA has been shown to decrease with weight loss [27], [28]. ADMA is higher in women with hypertension [29], obesity and pre-eclampsia [23], [30] [31], [32]. Supplementation with arginine or l-citrulline (which is metabolised to arginine) improves vascular function by increasing the arginine/ADMA ratio thereby increasing NO availability [33]. A recent systematic review [34] reported encouraging benefits of l-arginine supplementation in pregnant women with established hypertensive disease. More recently, l-Citrulline treatment in mid pregnancy has also been associated with improved vascular and angiogenic profiles in obese pregnant women [35].

Box 1 **Mechanisms linking obesity and preeclampsia**

• Insulin resistance
• ↑Leptin which correlates with insulin resistance but also has cytokine functions and activates monocytes
• ↓Adiponectin with reduced insulin sensitivity and reduced fatty acid oxidation
• Altered lipids and FFA
• Pro-oxidant state
• Altered adipose tissue response to the metabolic challenge of pregnancy
• Altered baseline angiogenic state (outside pregnancy)
• Chronic low level inflammation – subtle increase in CRP
• ↑ADMA levels

Alt-text: Box 1

### 2.3 Obesity and other cardiovascular disease - potential clues to mechanism?

As highlighted above, there is obviously considerable overlap in the metabolic features associated with obesity and the risk of preeclampsia and cardiovascular disease; it would therefore seem reasonable to interrogate the cardiovascular and metabolic syndrome literature to identify possible pathophysiological mechanisms which link obesity to preeclampsia risk. One such example is a study by Fabbrini et al. [36] in which obese individuals with either normal or abnormal metabolic profiles, defined by intra hepatic triglyceride (IHTG) content, were subjected to a metabolic challenge in the form of weight gain. The hypothesis was that obese individuals, with a normal metabolic profile, would be resistant to a weight gain challenge and there would be a difference in the adipose tissue response between the groups. In the metabolically abnormal group moderate weight gain exacerbated several metabolic risk factors for cardiovascular disease, including increased blood pressure, plasma triglyceride levels and VLDL apoB100, and decreased plasma adiponectin concentrations and insulin sensitivity in the liver, skeletal muscle and adipose tissues. It therefore seems plausible that obese women with abnormal metabolic health at the beginning of pregnancy are most likely to develop preeclampsia. Efforts to identify metabolic ill health using available markers [37] and biophysical characteristics may progress our understanding of the relationship between obesity and preeclampsia.

## 3 Chronic hypertension

In a systematic review examining pregnancy outcomes amongst women with chronic hypertension, the incidence of preeclampsia was 26% [38]. In absolute terms this makes chronic hypertension the strongest risk factor for preeclampsia. Conversely, it is also the risk factor least well studied; a result of the fact that several different underlying conditions contribute to a diagnosis of prepregnancy hypertension, which may independently contribute to the risk of preeclampsia through different underlying mechanisms. In a prospective study by Chappell et al. the risk factors identified as being associated with preeclampsia amongst women with chronic hypertension were black ethnicity, obesity and smoking [39]. Women with chronic hypertension appear to be at particularly high risk of developing preterm preeclampsia, commonly associated with fetal growth restriction (FGR). This observation would suggest that in women with chronic hypertension early placentation is often abnormal, possibly as a result of chronic changes in the maternal vascular endothelium including altered levels of inflammation and oxidative stress. Alternatively, increased resistance within the small vessels, reflective of increased systemic pressure, may cause damage to the developing placenta and increase the risk of preeclampsia and FGR. This hypothesis, however, is very difficult to test and can only be inferred by indirect evidence that elevated blood pressure in the first trimester in women with chronic hypertension is a consistent risk factor [39]. Given the very high risk of preeclampsia in women with chronic hypertension, its increasing prevalence and the overlap with older maternal age, obesity and the metabolic syndrome, further research investigating the underlying mechanisms that lead to preeclampsia in this group should be a high priority. In keeping with our increased knowledge of the heterogeneity of preeclampsia, attention to the different mechanistic pathways leading to hypertension may reveal important pathways relevant to the development of preeclampsia.

## 4 Maternal age

A recent meta analysis of 38 studies investigating the impact of advanced maternal age on pregnancy outcome included data from over 10 million women with an overall preeclampsia rate of 3.2% [40]. There was a large degree of heterogeneity between the studies, but a consistent increase in risk with increasing age was demonstrated with an overall OR for >35 years of 1.99 (95% CI 1.66–2.36). The mechanism by which older maternal age contributes to an increased risk of preeclampsia is poorly understood and most importantly the independent effect of age over the presence of other comorbidities more frequent in older women, has not been satisfactorily explored. Future research which is able to dissect out the factors associated with advanced maternal age that directly contribute to the risk of preeclampsia, over and above obesity and hypertension, would provide another significant advance.

## 5 ART/nulliparity

Nulliparity is the largest population attributable risk factor for preeclampsia [4] and several theories have suggested causal links, many of which relate to an immunological mechanism [41]. More recently, data have emerged from the assisted reproduction literature providing additional epidemiological confirmation that perhaps ‘foreign’ antigen increases the risk of preeclampsia, with the incidence of preeclampsia being higher in women conceiving after oocyte donation compared to women conceiving with other assisted reproductive techniques [42]. The techniques associated with in vitro fertilisation may also influence risk. For example, in a study of over 300,000 pregnancies from the CoNARTS cohort across Sweden, Denmark and Norway there was a modest increase in the number of pregnancies complicated by hypertensive disease (4.7–5.9%) [43] following IVF treatment. The risk was further modified by the use of a fresh embryo transfer in comparison to a cycle using a frozen embryo. In repeated cycles, frozen-frozen was associated with no change in risk, but in women receiving a frozen embryo followed by fresh embryo there was a significant reduction in the risk of preeclampsia, which was reversed in women who had a fresh cycle followed by frozen embryo transfer. These data suggest that very early events related either to the embryo, the intrauterine environment and/or hormonal status influence the risk of developing preeclampsia. In concordance with older maternal age and chronic hypertension, the risk of preeclampsia associated with assisted reproduction appears to be amplified by maternal obesity. In a study of over 10,000 pregnancies (348 with IVF) [44], the risk of preeclampsia was significantly higher in obese women following IVF. In interaction analysis, there was evidence of departure from multiplicativity which suggests that a high BMI interacts with IVF, although an additive effect of modification was not confirmed. Using detailed data from assisted reproduction datasets and exploring the interactions between maternal factors, and assisted reproduction techniques has the potential to identify important mechanistic links for the development of preeclampsia.

## 6 Interaction and causality

One of the unanswered questions regarding the epidemiology of preeclampsia is an explanation for the discrepancy in risk of recurrence in subsequent pregnancies. Whilst a history of preeclampsia is a strong risk factor and the risk of recurrence is around 25% [45], more women do not develop preeclampsia in a subsequent pregnancy than do. This is despite many of the traditional risk factors becoming more prevalent in subsequent pregnancies, including older age, increasing BMI and worsening hypertension resulting in worse metabolic health. The interaction between recurrent disease and BMI has been investigated and in one study using registry data in Utah, obesity appeared to have a stronger association with incident preeclampsia in first or second pregnancies but was less strongly associated with recurrent preeclampsia [46]. This suggests that the pathophysiology of recurrent preeclampsia may be different to isolated cases occurring in the presence of risk factors such as obesity.

In summary it is clear that obesity, poor metabolic and vascular health have a strong associations with the development of preeclampsia. These risk factors are potentially amplified by assisted reproductive techniques and other risk factors such as ethnicity and maternal age. These risk factors have been consistently reported and now future epidemiological and mechanistic studies need to focus on studying potential mechanisms within at risk groups (e.g. poor metabolic health amongst obese women) and the interaction between risk factor (e.g. IVF in high risk groups) to further progress our understanding of the pathophysiology of this condition.

## Funding

JM is supported by a NIHR Clinician Scientist fellowship (NIHR-CS-011-020).

## References

[1] Wallis A.B., Saftlas A.F., Hsia J., Atrash H.K. (2008). Secular trends in the rates of preeclampsia, eclampsia, and gestational hypertension, United States, 1987-2004. Am. J. Hypertens..

[2] Dahlstrom B.L., Engh M.E., Bukholm G., Oian P. (2006). Changes in the prevalence of pre-eclampsia in Akershus County and the rest of Norway during the past 35 years. Acta obstetricia Gynecol. Scand..

[3] Hutcheon J.A., Lisonkova S., Joseph K.S. (2011). Epidemiology of pre-eclampsia and the other hypertensive disorders of pregnancy, Best practice & research. Clin. obstetrics Gynaecol..

[4] Bartsch E., Medcalf K.E., Park A.L., Ray J.G. (2016). Clinical risk factors for pre-eclampsia determined in early pregnancy: systematic review and meta-analysis of large cohort studies. BMJ.

[5] Bilano V.L., Ota E., Ganchimeg T., Mori R., Souza J.P. (2014). Risk factors of pre-eclampsia/eclampsia and its adverse outcomes in low- and middle-income countries: a WHO secondary analysis. PloS one.

[6] Kenny L.C., Black M.A., Poston L., Taylor R., Myers J.E., Baker P.N., McCowan L.M., Simpson N.A., Dekker G.A., Roberts C.T., Rodems K., Noland B., Raymundo M., Walker J.J., North R.A. (2014). Early pregnancy prediction of preeclampsia in nulliparous women, combining clinical risk and biomarkers: the screening for pregnancy endpoints (SCOPE) international cohort study. Hypertension.

[7] Bodnar L.M., Catov J.M., Klebanoff M.A., Ness R.B., Roberts J.M. (2007). Prepregnancy body mass index and the occurrence of severe hypertensive disorders of pregnancy. Epidemiology.

[8] Mbah A.K., Kornosky J.L., Kristensen S., August E.M., Alio A.P., Marty P.J., Belogolovkin V., Bruder K., Salihu H.M. (2010). Super-obesity and risk for early and late pre-eclampsia. BJOG An Int. J. Obstet. Gynaecol..

[9] Jeyabalan A. (2013). Epidemiology of preeclampsia: impact of obesity. Nutr. Rev..

[10] Kaaja R. (1998). Insulin resistance syndrome in preeclampsia. Seminars reproductive Endocrinol..

[11] Bodnar L.M., Ness R.B., Harger G.F., Roberts J.M. (2005). Inflammation and triglycerides partially mediate the effect of prepregnancy body mass index on the risk of preeclampsia. Am. J. Epidemiol..

[12] Wolf M., Kettyle E., Sandler L., Ecker J.L., Roberts J., Thadhani R. (2001). Obesity and preeclampsia: the potential role of inflammation. Obstetrics Gynecol..

[13] Vieira M.C., Poston L., Fyfe E., Gillett A., Kenny L.C., Roberts C.T., Baker P.N., Myers J.E., Walker J.J., McCowan L.M., North R.A., Pasupathy D. (2017). Clinical and biochemical factors associated with preeclampsia in women with obesity. Obes. Silver Spring.

[14] Myers J.E., Kenny L.C., McCowan L.M., Chan E.H., Dekker G.A., Poston L., Simpson N.A., North R.A. (2013). Angiogenic factors combined with clinical risk factors to predict preterm pre-eclampsia in nulliparous women: a predictive test accuracy study. BJOG An Int. J. Obstet. Gynaecol..

[15] Dewerchin M., Carmeliet P. (2012). PlGF: a multitasking cytokine with disease-restricted activity. Cold Spring Harb. Perspect. Med..

[16] Mijal R.S., Holzman C.B., Rana S., Karumanchi S.A., Wang J., Sikorskii A. (2011). Midpregnancy levels of angiogenic markers in relation to maternal characteristics. Am. J. obstetrics Gynecol..

[17] Ghosh S.K., Raheja S., Tuli A., Raghunandan C., Agarwal S. (2013). Serum placental growth factor as a predictor of early onset preeclampsia in overweight/obese pregnant women. J. Am. Soc. Hypertens. JASH.

[18] Faupel-Badger J.M., Staff A.C., Thadhani R., Powe C.E., Potischman N., Hoover R.N., Troisi R. (2011). Maternal angiogenic profile in pregnancies that remain normotensive. Eur. J. obstetrics, Gynecol. reproductive Biol..

[19] Correia M.L., Haynes W.G. (2004). Leptin, obesity and cardiovascular disease. Curr. Opin. Nephrol. Hypertens..

[20] Teppa R.J., Ness R.B., Crombleholme W.R., Roberts J.M. (2000). Free leptin is increased in normal pregnancy and further increased in preeclampsia. Metabolism Clin. Exp..

[21] Mazaki-Tovi S., Romero R., Vaisbuch E., Kusanovic J.P., Erez O., Gotsch F., Chaiworapongsa T., Than N.G., Kim S.K., Nhan-Chang C.L., Jodicke C., Pacora P., Yeo L., Dong Z., Yoon B.H., Hassan S.S., Mittal P. (2009). Maternal serum adiponectin multimers preeclampsia. J. Perinat. Med..

[22] Giacchetti G., Faloia E., Sardu C., Camilloni M.A., Mariniello B., Gatti C., Garrapa G.G., Guerrieri M., Mantero F. (2000). Gene expression of angiotensinogen in adipose tissue of obese patients. Int. J. Obes. Relat. Metab. Disord..

[23] Dandona P., Aljada A., Chaudhuri A., Mohanty P., Garg R. (2005). Metabolic syndrome: a comprehensive perspective based on interactions between obesity, diabetes, and inflammation. Circulation.

[24] Poston L., Chappell L.C. (2001). Is oxidative stress involved in the aetiology of pre-eclampsia?. Acta Paediatr. Suppl..

[25] Roberts J.M., Hubel C.A. (1999). Is oxidative stress the link in the two-stage model of pre-eclampsia?. Lancet.

[26] Boger R.H., Bode-Boger S.M. (2000). Asymmetric dimethylarginine, derangements of the endothelial nitric oxide synthase pathway, and cardiovascular diseases. Seminars thrombosis hemostasis.

[27] Eid H.M., Arnesen H., Hjerkinn E.M., Lyberg T., Seljeflot I. (2004). Relationship between obesity, smoking, and the endogenous nitric oxide synthase inhibitor, asymmetric dimethylarginine. Metabolism Clin. Exp..

[28] Krzyzanowska K., Mittermayer F., Kopp H.P., Wolzt M., Schernthaner G. (2004). Weight loss reduces circulating asymmetrical dimethylarginine concentrations in morbidly obese women. J. Clin. Endocrinol. metabolism.

[29] Sonmez A., Celebi G., Erdem G., Tapan S., Genc H., Tasci I., Ercin C.N., Dogru T., Kilic S., Uckaya G., Yilmaz M.I., Erbil M.K., Kutlu M. (2010). Plasma apelin and ADMA Levels in patients with essential hypertension. Clin. Exp. Hypertens..

[30] Savvidou M.D., Hingorani A.D., Tsikas D., Frolich J.C., Vallance P., Nicolaides K.H. (2003). Endothelial dysfunction and raised plasma concentrations of asymmetric dimethylarginine in pregnant women who subsequently develop pre-eclampsia. Lancet.

[31] Boger R.H., Diemert A., Schwedhelm E., Luneburg N., Maas R., Hecher K. (2010). The role of nitric oxide synthase inhibition by asymmetric dimethylarginine in the pathophysiology of preeclampsia. Gynecol. obstetric investigation.

[32] Speer P.D., Powers R.W., Frank M.P., Harger G., Markovic N., Roberts J.M. (2008). Elevated asymmetric dimethylarginine concentrations precede clinical preeclampsia, but not pregnancies with small-for-gestational-age infants. Am. J. obstetrics Gynecol..

[33] Ochiai M., Hayashi T., Morita M., Ina K., Maeda M., Watanabe F., Morishita K. (2012). Short-term effects of L-citrulline supplementation on arterial stiffness in middle-aged men. Int. J. Cardiol..

[34] Dorniak-Wall T., Grivell R.M., Dekker G.A., Hague W., Dodd J.M. (2014). The role of L-arginine in the prevention and treatment of pre-eclampsia: a systematic review of randomised trials. J. Hum. Hypertens..

[35] Powers R., Weissgerber T.L., McGonigal S., Myerski A., Gallaher M., Speer P.D., Roberts J.M., Jeyabalan A., Hubel C.A. (2015). [7-OR]: L-Citrulline administration increases the arginine/ADMA ratio, decreases blood pressure and improves vascular function in obese pregnant women. Pregnancy Hypertens..

[36] Fabbrini E., Yoshino J., Yoshino M., Magkos F., Tiemann Luecking C., Samovski D., Fraterrigo G., Okunade A.L., Patterson B.W., Klein S. (2015). Metabolically normal obese people are protected from adverse effects following weight gain. J. Clin. investigation.

[37] Phillips C.M., Perry I.J. (2013). Does inflammation determine metabolic health status in obese and nonobese adults?. J. Clin. Endocrinol. metabolism.

[38] Bramham K., Parnell B., Nelson-Piercy C., Seed P.T., Poston L., Chappell L.C. (2014). Chronic hypertension and pregnancy outcomes: systematic review and meta-analysis. BMJ.

[39] Chappell L.C., Enye S., Seed P., Briley A.L., Poston L., Shennan A.H. (2008). Adverse perinatal outcomes and risk factors for preeclampsia in women with chronic hypertension: a prospective study. Hypertension.

[40] Lean S., Derricott H., Jones R., Heazell A. (2016). Systematic review and meta-analysis of stillbirth and related complications in advanced maternal age provides a rationale for focussing on placental dysfunction in these pregnancies. Reprod. Sci..

[41] Redman C.W. (1991). Immunology of preeclampsia. Semin. Perinatol..

[42] Masoudian P., Nasr A., de Nanassy J., Fung-Kee-Fung K., Bainbridge S.A., El Demellawy D. (2016). Oocyte donation pregnancies and the risk of preeclampsia or gestational hypertension: a systematic review and metaanalysis. Am. J. obstetrics Gynecol..

[43] Opdahl S., Henningsen A.A., Tiitinen A., Bergh C., Pinborg A., Romundstad P.R., Wennerholm U.B., Gissler M., Skjaerven R., Romundstad L.B. (2015). Risk of hypertensive disorders in pregnancies following assisted reproductive technology: a cohort study from the CoNARTaS group. Hum. Reprod..

[44] Dayan N., Pilote L., Opatrny L., Basso O., Messerlian C., El-Messidi A., Daskalopoulou S.S. (2015). Combined impact of high body mass index and in vitro fertilization on preeclampsia risk: a hospital-based cohort study. Obes. Silver Spring.

[45] Dildy G.A., Belfort M.A., Smulian J.C. (2007). Preeclampsia recurrence and prevention. Semin. Perinatol..

[46] Boghossian N.S., Yeung E., Mendola P., Hinkle S.N., Laughon S.K., Zhang C., Albert P.S. (2014). Risk factors differ between recurrent and incident preeclampsia: a hospital-based cohort study. Ann. Epidemiol..

